# Early hematological indices as a predictor of placenta accreta in women with high suspicion of accreta

**DOI:** 10.1097/MD.0000000000041084

**Published:** 2025-01-03

**Authors:** Ali Mohammed Ali Al-Nuaimi, Zahraa Mohammed Ali Al-Nuaimi

**Affiliations:** a College of Pharmacy, Gilgamesh University, Baghdad, Iraq; b Department of Gynecology and Obstetrics, Al-Yarmouk Teaching Hospital, Baghdad, Iraq.

**Keywords:** early predictors, lymphocyte, placenta accreta, platelet, red cell distribution width

## Abstract

There is a lack of early biomarkers to predict the placenta accreta spectrum; thus, searching for available and easily obtained markers such as hematological indices is an attractive option. The current study is a diagnostic accuracy study included 198 women; all women underwent an assessment of their hematological indices during their first trimester as part of their routine antenatal care. All women included in the study had a high suspicion of developing placenta accreta spectrum; the women were followed up until their delivery. White blood cell, neutrophil count, and red cell distribution width (RDW) were significantly higher in the women with accreta than those without accreta. RDW had fair ability (area under the curve, 0.707) as a predictor of accreta. RDW had the highest positive and lowest negative likelihood ratios (indicating better value than the rest of the markers). In multivariate analysis, RDW and the platelet-lymphocyte ratio were independently associated with accreta after adjustment to the effects of age, gestational age, gravida, parity, abortion, and past medical/surgical history. In conclusion, simple, early blood count parameters may be utilized for placenta accreta; RDW appears to be the best predictor of placenta accreta.

## 1. Introduction

During an ordinary pregnancy, the placenta securely attaches to the endometrium that has undergone decidualization.^[1]^ Placenta accreta refers to the atypical infiltration of placental trophoblasts into the uterine myometrium. The classification of illnesses related to the degree of myometrial invasion includes placenta accreta, placenta increta, and placenta percreta. Placenta accreta spectrum (PAS) diseases are linked to higher rates of maternal morbidity and mortality.^[2,3]^ Anomalous adhesion and invasion of villous tissue into the myometrium lead to the placenta’s inability to detach naturally from the uterine wall after birth.^[4,5]^ Unexpectedly, when trying to remove accreta villous tissue during birth physically, it often causes immediate bleeding from the uteroplacental circulation.^[6,7]^ In instances where the condition becomes invasive, it might result in significant obstetric hemorrhage as a result of the disturbance to the deep uterine blood vessels in the increta or percreta region. Unsurprisingly, the use of prenatal diagnosis for PAS has been demonstrated to reduce maternal illness and death, making it crucial for improving the management of the condition.^[8,9]^ The incidence of PAS in research published from 1982 to 2018 varied from 1 in 100 to 1 in 10,000. The prevalence of PAS has been on the rise in recent decades; this can be linked to factors such as a history of cesarean section (CS), previous cases of placenta accreta, placenta previa (PP), advanced maternal age, pregnancies resulting from in vitro fertilization, and maternal obesity.^[10–13]^

In recent decades, extensive epidemiological research has revealed the impact of the significant rise in cesarean delivery rates on the risks associated with PAS.^[14,15]^ Women who have significant medical risk indicators for PAS, such as PP, previous cesarean delivery, endometrial ablation, or other uterine surgery, should receive a diagnostic assessment from a practitioner who specializes in this disorder.^[16,17]^ Obstetrical sonography during the second or third trimester of pregnancy is the primary method for diagnosing conditions related to PAS.^[18,19]^ The disorder is sometimes diagnosed in the initial 3 months of pregnancy, usually by detecting an ectopic pregnancy.^[6]^

Different types of immune cells, including natural killer cells, macrophages, T cells, B cells, and dendritic cells, are found at the maternal-fetal interface. Macrophages in the decidua regulate trophoblast invasion, artery remodeling, and the onset of labor and recovery of the uterus after childbirth.^[20]^ The ratio of M − 1 to M − 2 macrophages at the maternal-fetal contact plays a vital role in pregnancy. Furthermore, T cells make up around 10% of all immune cells in the decidua during the first trimester.^[20]^ These T cells play a crucial role in suppressing the immune response and are characterized by the expression of CD^4+^CD^25+^ markers. The presence of regulatory T cells in the peripheral blood and decidua of abortuses was dramatically reduced. PAS is characterized by an anomaly in the development of blood vessels in the placenta, known as placental angiogenesis. During pregnancy, the successful invasion of trophoblast cells and the formation of the placenta rely on the precise production of certain crucial regulators.^[20]^

A cesarean birth scar may potentially impact the recruitment of leukocytes to the endometrium during the secretory period. A study on uterine circulation in women who have had a previous cesarean delivery found that there is a rise in uterine vascular resistance and a decline in blood flow volume compared with women who have had a previous vaginal birth^[21,22]^; this implies that blood flow is hindered near the scar. Inadequate blood supply to the scar region can result in or contribute to long-lasting localized deterioration of the uterine muscle and diminished or nonexistent regrowth of the scar tissue.^[21]^

While various biomarkers, including increased levels of maternal serum alpha-fetoprotein, placental pregnancy-associated plasma protein A, and pro-brain natriuretic peptide, have been linked to PAS, none of them have been demonstrated to be clinically predictive.^[23,24]^ Yet, the search continues to find easily obtained and reliable markers for PAS. Recently, some of the blood parameters have gained popularity in diagnosing some gynecological disorders such as preeclampsia,^[25–28]^ endometrial cancer,^[29]^ gestational trophoblastic disease,^[30]^ hyperemesis gravidarum,^[31]^ ectopic pregnancy,^[32]^ and preterm birth.^[33]^ These blood parameters are poorly examined in PAS; as such, this study examined the diagnostic potential of some blood cell parameters in the first and second trimesters to predict the development of PAS.

## 2. Methods

### 2.1. Study design

In a diagnostic accuracy study that included 198 women, women underwent an assessment of their hematological indices during their first trimester as patients of their routine antenatal care. All women included in the study had high suspicion of developing PAS (previous CS operation with complete PP); the women were follow-up till their delivery.

The PP and accreta were diagnosed using ultrasonography and Doppler studies (Mindray DC N3 Pro; a transabdominal approach was done using a 5-MHz 3C5A probe). The diagnostic criteria for identifying PAS with ultrasound include the absence of the echo-free space behind the placenta, irregularity in the connection between the uterus and placenta, and a weakening of the uterine muscle of <1 mm. The diagnosis of PAS was verified after delivery and confirmed histopathologically.^[34]^

### 2.2. Study settings

The data were collected from the Department of Obstetrics and Gynecology, Al-Yarmouk Teaching Hospital, between October 2023 and July 2024. Written informed consent was obtained from all women who participated in the study.

### 2.3. Ethical approval

The study was approved by the College of Pharmacy, Gilgamesh University Research Ethical Committee (approval number: GAU-2023-004; date: October 2, 2023), and written informed consent was obtained from all participants, per the Helsinki Declaration and its later amendments.

### 2.4. Inclusion criteria

Women aged more than or equal to 18 years, previous CS with complete PP, singleton delivery, and living fetus.

### 2.5. Exclusion criteria

Women who have experienced ruptured membranes, vaginal bleeding, chorioamnionitis, multiple pregnancies, or any medical condition, as well as those with infections or medical problems that may impact their full blood picture characteristics.

### 2.6. Clinical assessment

At the first presentation, the authors interviewed and examined each woman as part of their antenatal care and assessment of the women for eligibility criteria. A transabdominal ultrasound was performed to confirm the presence of PP. If the women have placenta accreta and a previous cesarean section, written consent was obtained. The authors obtained detailed information, including maternal and gestational age, gynecological history, and past medical and surgical history. A maternal blood sample was collected during the presentation (before intervention and before receiving any medication) and sent to the laboratory analysis of the hematological indices (ADVIA 2120i, Siemens Healthcare, Germany). Five milliliters of venous blood were collected from each woman and placed in sterile tubes labeled with the name of a patient and centrifuged for 15 minutes and examined for white blood cell (WBC), neutrophil, lymphocyte, platelet, hematocrit, hemoglobin, mean platelet volume (MPV), and red blood cell distribution width (RDW); the analysis carried out in the laboratory department in Al-Yarmouk Teaching Hospital.

### 2.7. Sample size

Based on the web calculator for cohort studies (https://epitools.ausvet.com.au/cohortss), assuming that the incidence of PAS in Iraqi women was 1.4% of total deliveries.^[3]^ Assuming a relative risk of 5, a 95% confidence interval, and an 80% power of detection, we arrived at a minimum sample size of 198 women.

### 2.8. Statistical analysis

The Anderson-Darling test was used to assess whether the data followed the Gaussian distribution; for the parametric variable, an independent *t* test was used; for nonparametric parameters, the Mann-Whitney *U* test was used, median and interquartile ranges were used to present the data, receiver operating characteristic curve analysis was used to assess the diagnostic validity of the hematological parameters to predict accreta, all analysis was done using GraphPad Prism 10.3, and the *P* value was considered significant if ≤.05.

## 3. Results

One hundred ninety-eight women with PP completed the study; 75 (37.9%) developed placenta accreta, while 123 (62.1%) did not develop placenta accreta. Mean maternal age and gestational age at delivery of accreta patients were significantly higher than those in the no accreta group. There was no significant difference in gravida, parity, abortion, and past gynecological history between control and accreta, as seen in Table 1.

**Table 1:** Baseline characteristics.

Variables	No accreta	Accreta	*P* value
Number	123	75	
Age (yr), mean ± SD	29.9 ± 5.9	32.0 ± 5.6	.014
Gestational age at delivery (wk), mean ± SD	36.2 ± 2.5	35.1 ± 2.6	.003
Gravida, mean ± SD	5.0 ± 2.4	5.1 ± 1.1	.631
Parity, mean ± SD	3.3 ± 1.7	3.4 ± 1.1	.472
Abortion, median (IQR)	0 (0–1)	0 (0–1)	.939
Past gynecological surgical history, n (%)			.960
No history	75 (61.0%)	46 (61.3%)	
Positive history	48 (39.0%)	29 (38.7%)	

IQR = interquartile range, SD = standard deviation.

**Figure 1.**
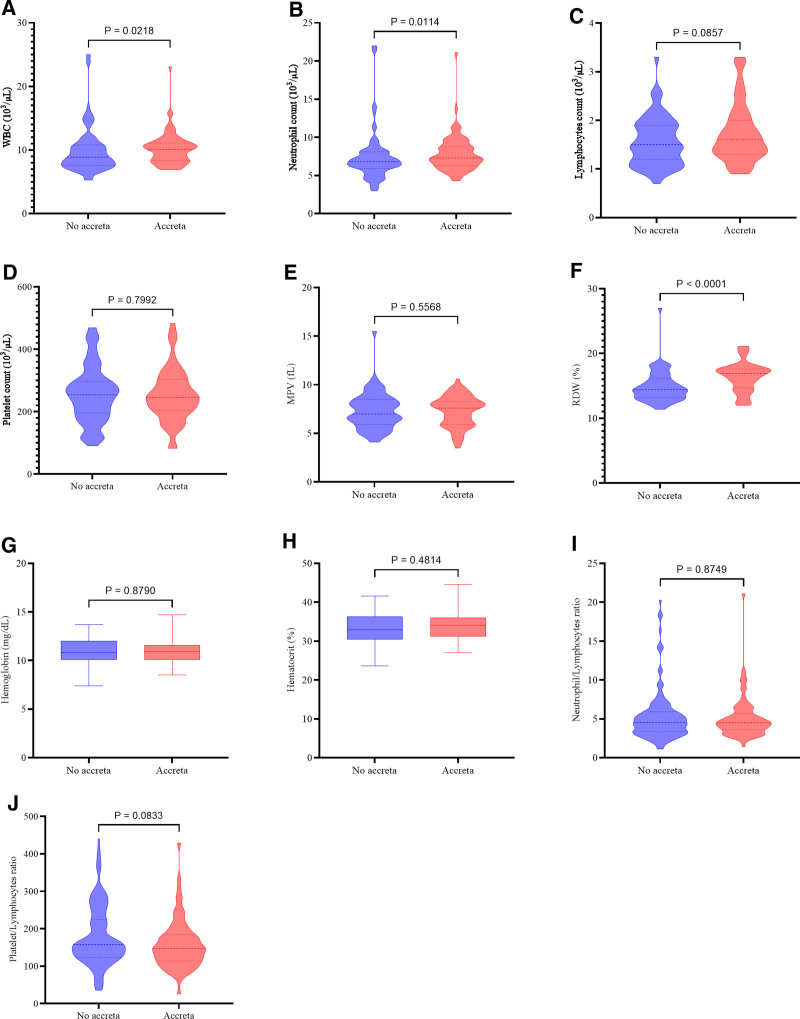
Assessment of hematological parameters according to the presence of placenta accreta. (A) White blood cell count (WBC), (B) neutrophil count, (C) lymphocyte count, (D) platelet count, (E) mean platelet volume (MPV), (F) red cell distribution width (RDW), (G) hemoglobin, (H) hematocrit, (I) neutrophil/lymphocyte ratio, and (J) platelet/lymphocyte ratio.

WBC, neutrophil count, and red cell distribution width (RDW) were significantly higher in the women with accreta compared with those without accreta; the rest of the hematological parameters did not show a significant difference, as seen in Figure 1 and Table 2.

**Table 2:** Descriptive value of the hematological parameters according to study groups.

Variables	No accreta	Accreta
Number	123	75
WBC count (×10^3^/µL), median (IQR)	8.9 (7.6–10.8)	10.1 (8.4–11.1)
Neutrophil count (×10^3^/µL), median (IQR)	6.8 (5.9–8.1)	7.3 (6.3–8.8)
Lymphocyte count (×10^3^/µL), median (IQR)	1.5 (1.2–1.9)	1.6 (1.3–2)
Platelet count (×10^3^/µL), median (IQR)	254 (196–298)	246 (204–304)
MPV (fL), median (IQR)	7 (5.9–8.5)	7.6 (5.9–8.3)
RDW (%), median (IQR)	14.4 (13.2–16.1)	16.9 (14.7-17.6)
NLR, median (IQR)	4.556 (3.333–5.917)	4.5 (3.647–5.727)
PLR, median (IQR)	224.7 (157.5–440)	183.8 (147–429)
Hemoglobin (mg/dL), mean ± SD	10.87 ± 1.433	10.90 ± 1.232
Hematocrit (%), mean ± SD	33.18 ± 4.130	33.58 ± 3.345

IQR = interquartile range, MPV = mean platelet volume, NLR = neutrophil-lymphocyte ratio, PLR = platelet-lymphocyte ratio, RDW = red cell distribution width, SD = standard deviation, WBC = white blood cell.

**Figure 2.**
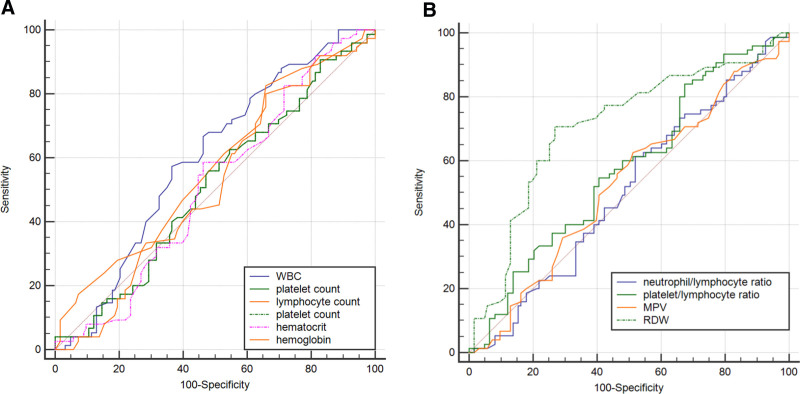
Receiver operating characteristic curve of the predictors of accreta. (A) White blood cell count, neutrophil count, lymphocyte count, platelet count, hemoglobin, and hematocrit. (B) Mean platelet volume (MPV), red cell distribution width (RDW), neutrophil/lymphocyte ratio, and platelet/lymphocyte ratio.

**Figure 3.**
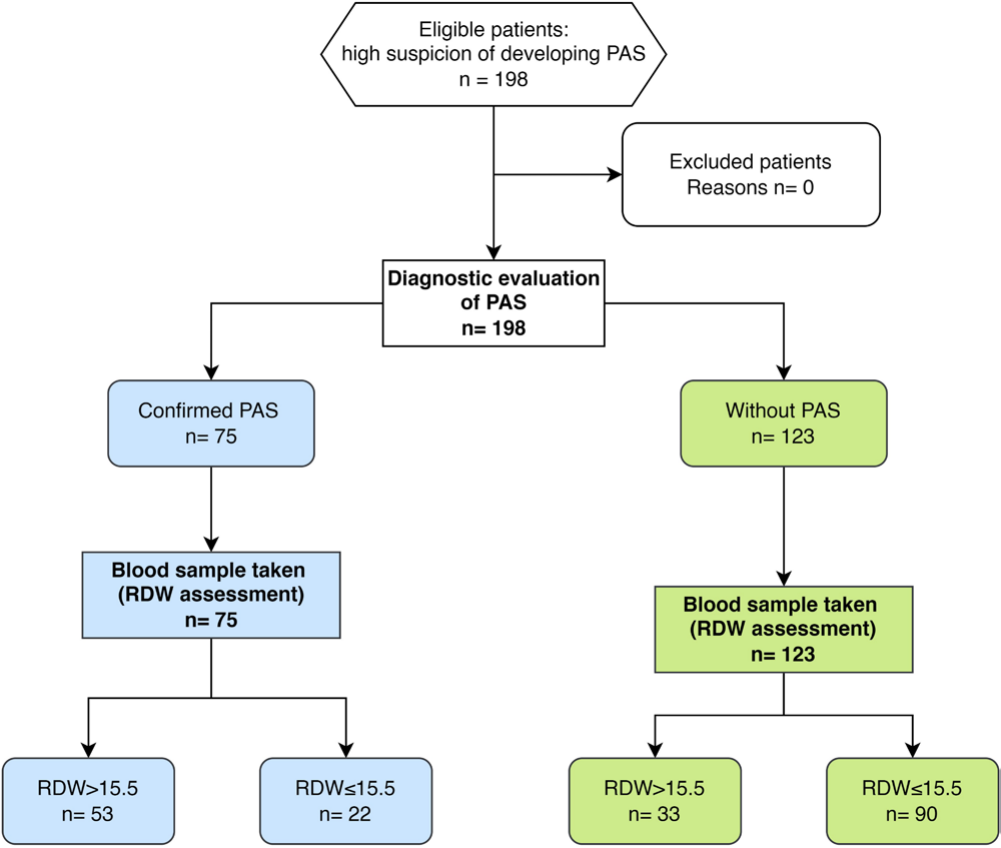
STARD flowchart of the study. PAS = placenta accreta spectrum, RDW = red cell distribution width, STARD = Standards for Reporting Diagnostic accuracy studies.

RDW had fair ability (since the area under the curve [AUC] is between 0.7 and 0.79) as a predictor of accreta. In contrast, the platelet/lymphocyte (P/L) ratio had a poor ability to predict accreta (since the AUC < 0.7), and RDW had the highest positive likelihood ratio (LH) and the lowest negative LH (indicating a better value than the rest of the markers). Regarding sensitivity (SN) and specificity (SP), RDW showed moderate SN and SP (70.67 and 73.17%). The P/L ratio showed higher SN (84%) but poor SP (32.52%), as illustrated in Table 3 and Figure 2. The Standards for Reporting Diagnostic accuracy studies flowchart of RDW is illustrated in Figure 3.

**Table 3:** Diagnostic validity and clinical utility of the predictors of accreta.

	AUC	95% CI of AUC	+LH	−LH	*P* value
RDW	0.707	0.638–0.769	2.63	0.40	<.001
P/L ratio	0.573	0.501–0.643	1.24	0.49	.077

	Cutoff	SN	SP	PPV	NPV
RDW	>15.5	70.67	73.17	61.6	80.4
P/L ratio	≤202.6667	84.00	32.52	43.2	76.9

AUC = area under the curve, CI = confidence interval, LH = likelihood ratio, NPV = negative predictive value, P/L = platelet/lymphocyte, PPV = positive predictive value, RDW = red cell distribution width, SN = sensitivity, SP = specificity.

In multivariate analysis, RDW and the platelet-lymphocyte ratio (PLR) were independently associated with accreta after adjustment to the effects of age, gestational age, gravida, parity, abortion, and past medical/surgical history, as illustrated in Table 4.

**Table 4:** Multivariate analysis of the predictors of accreta.

	Wald	OR	95% CI	*P* value
WBC count	0.821	0.953	0.858–1.058	.365
Neutrophil count	0.168	0.977	0.876–1.090	.682
RDW	17.158	1.384	1.187–1.614	<.001
P/L ratio	6.219	0.994	0.990–0.999	.013

CI = confidence interval, OR = odds ratio, R/L ratio = platelet/lymphocyte ratio, RDW = red cell distribution width, WBC = white blood cell.

## 4. Discussion

Anisocytosis (presented by red blood cell distribution width [RDW]) and changes in other blood cell parameters have been associated with the severity and prognosis of several acute and chronic diseases, as well as physiological conditions such as pregnancy, miscarriage, gestational diabetics, preeclampsia, and recently placental invasion diseases.^[35]^

In the present study, we examined the predictive ability of first-trimester complete blood count parameters in predicting the later development of placenta accreta; several parameters showed good promise as predictors (total WBC, neutrophil count, and RDW), but only RDW was presented with sufficient diagnostic validity with AUC > 0.7. In addition, RDW showed an independent relationship with placenta accreta after adjustment to the effects of age, gestational age, gravida, parity, abortion, and past medical/surgical history; this can be explained by the fact that maternal circulating leukocytes are activated in pregnancy and further activated in PAS (since it is related to increased inflammation that occurred in PAS), in which these activated leukocytes could be responsible for the vascular dysfunction associated with PAS.^[36]^

In addition, RDW showed moderate SN and SP (70.67 and 73.17%). The P/L ratio showed higher SN (84%) but poor SP (32.52%). Regarding posterior probability, RDW showed a good negative predictive value (80.4%) and low positive predictive value (61.5%), which suggests that RDW is a good screening tool, particularly in excluding conditions other than PAS.^[37]^ These results indicate that RDW is ideal as an early screening tool for PAS, owing to its high negative predictive value.^[38]^

RDW is characterized by high intrinsic plasticity of the external membrane and low hemoglobin content, which allow a certain degree of expansion or contraction in response to physiological or pathological stimuli.^[39]^ Higher RDW values reflect the presence of anisocytosis that may be attributable to small and large RBCs or both^[40]^; this indicates the elevation of RDW, reflected by the higher systemic inflammatory status in women with accreta.

In the present study, PLR was not significantly different in accreta compared with none accreta women, which was in agreement with the study by Yayla et al^[34]^ in which no significant difference between PAS and control group was found (117.9 ± 43.1 vs 122.1 ± 54.1). As seen in the present study, PLR is an inflammatory marker that is expected to be lower in diseases with elevated inflammatory status; however, it did not reach statistical significance.^[36]^

The present study showed no significant difference in MPV between both groups. In contrast, in the study by Yayla et al,^[34]^ MPV was significantly higher in PAS than control. Ersoy et al^[41]^ examined 93 PP women and compared them with 247 control women; they found that MPV was significantly lower in the PP group than the control. The variation in the effect of placenta accreta on MPV depends on the degree of inflammatory stress. In our patients, the degree of inflammatory stress was not strong enough to affect MPV; since we examined its effect early in pregnancy, further studies are required to examine its effect on the later trimester of pregnancy. These inconsistencies between studies required additional work to find the exact role of MPV in PAS diseases.

The neutrophil-lymphocyte ratio (NLR) was higher in women with accreta; however, it did not reach statistical significance. Our findings were in agreement with other studies such as the study by Yayla et al,^[34]^ in which NLR was significantly higher in PAS compared with control, and the study by Ersoy et al^[41]^ in which NLR was significantly higher in PP compared with control, despite the similarities between our studies and these studies.^[34,41]^ Our study did not reach statistical significance, which can be attributed to the difference in sample size, populations involved, and timing of CBC sampling.

In diagnostic SN analysis (using the receiver operating characteristic curve test), RDW showed the best results as a diagnostic tool for accreta, in which the AUC was highest and above 0.7; in addition, at cutoff > 15.5, it had positive LH = 2.63, indicating it increased the conformation of accreta by 20% to 25%, and at the same cutoff, it has negative LH = 0.40, which indicates that if used as a diagnostic tool, it will lead to 20% exclusion ability. Both of these findings indicate a similar ability of RDW if used as a diagnostic tool when incorporated into the diagnostic decision process for accreta. When we examined RDW validity as a single diagnostic test, it had similar SN (70.67%) and SP (73.13%); this indicates that RDW is a good test either as a single test or if incorporated with another test for diagnosis of accreta. There is a lack of studies that examine this part of RDW and its relationship with accreta. In the study by Yayla et al,^[34]^ RDW was also a good predictor of PAS (AUC, 0.718), which agrees with our findings.

In the study by Yayla et al,^[34]^ MPV was a good predictor of accreta (AUC, 0.807), which disagrees with our findings. MPV is considered to be a marker of platelet activation. Many studies examined this marker in inflammatory disease and found a direct association between MPV and inflammatory status, which explains the present study’s finding.

In multivariate analysis, RDW and PLR appear as independent predictors of accreta. In the study by Yayla et al,^[34]^ multivariate analyses revealed that age, MPV, RDW, and NLR were significantly associated with the PAS. This final analysis revealed similar findings between our results and the only available study similar to our work.

Multiple studies examined the role of blood cell parameters with tumors and other autoimmune diseases.^[42–44]^ Since tumors originate at sites of chronic inflammation,^[45]^ a programmed and controlled invasion of extravillous cytotrophoblasts into the decidua and the placental anchoring into the uterus are required for the functional placenta and, therefore, the healthy fetus.^[46]^ However, although this invasion is a well-controlled process, in some cases, excessive invasion of trophoblast into the decidua occurs, especially in cases with uterine scars where vascularization is presumably compromised^[47,48]^; this explains the novel association findings between placenta accreta and blood cell inflammatory parameters. Platelet activation is linked to the pathophysiology of diseases prone to thrombosis and inflammation. Therefore, several platelet markers, including MPV, have been investigated to determine the link between platelet activation and thrombosis and inflammation.^[49]^ However, no obvious relationship was found in this study.

## 5. Conclusion

Simple, early blood count parameters may be utilized for placenta placental accreta; RDW appears to be the best predictor of placenta accreta.

## Acknowledgments

The authors are grateful to Professor Shatha H. Ali for her guidance.

## Author contributions

**Conceptualization:** Ali Mohammed Ali Al-Nuaimi, Zahraa Mohammed Ali Al-Nuaimi.

**Data curation:** Ali Mohammed Ali Al-Nuaimi, Zahraa Mohammed Ali Al-Nuaimi.

**Formal analysis:** Ali Mohammed Ali Al-Nuaimi, Zahraa Mohammed Ali Al-Nuaimi.

**Funding acquisition:** Ali Mohammed Ali Al-Nuaimi, Zahraa Mohammed Ali Al-Nuaimi.

**Investigation:** Ali Mohammed Ali Al-Nuaimi, Zahraa Mohammed Ali Al-Nuaimi.

**Methodology:** Ali Mohammed Ali Al-Nuaimi, Zahraa Mohammed Ali Al-Nuaimi.

**Project administration:** Ali Mohammed Ali Al-Nuaimi, Zahraa Mohammed Ali Al-Nuaimi.

**Resources:** Ali Mohammed Ali Al-Nuaimi.

**Validation:** Ali Mohammed Ali Al-Nuaimi.

**Visualization:** Ali Mohammed Ali Al-Nuaimi, Zahraa Mohammed Ali Al-Nuaimi.

**Writing – original draft:** Ali Mohammed Ali Al-Nuaimi, Zahraa Mohammed Ali Al-Nuaimi.

**Writing – review & editing:** Ali Mohammed Ali Al-Nuaimi, Zahraa Mohammed Ali Al-Nuaimi.

**Supervision:** Zahraa Mohammed Ali Al-Nuaimi.
